# A Newfound Association between *MDC1* Functional Polymorphism and Lung Cancer Risk in Chinese

**DOI:** 10.1371/journal.pone.0106794

**Published:** 2014-09-08

**Authors:** Bo Wang, Lisha Zhang, Fuman Qiu, Wenxiang Fang, Jieqiong Deng, Yifeng Zhou, Jiachun Lu, Lei Yang

**Affiliations:** 1 The State Key Lab of Respiratory Disease, The Institute for Chemical Carcinogenesis, Guangzhou Medical University, Guangzhou, China; 2 Soochow University Laboratory of Cancer Molecular Genetics, Medical College of Soochow University, Suzhou, China; IPO, Inst Port Oncology, Portugal

## Abstract

Mediator of DNA damage checkpoint protein 1 (MDC1) plays an early and core role in Double-Strand Break Repair (DDR) and ataxia telangiectasia-mutated (ATM) mediated response to DNA double-strand breaks (DSBs), and thus involves the pathogenesis of several DNA damage-related diseases such as cancer. We hypothesized that the single nucleotide polymorphisms (SNPs) of *MDC1* which have potencies on affecting MDC1 expression or function were associated with risk of lung cancer. In a two-stage case-control study, we tested the association between 5 putatively functional SNPs of *MDC1* and lung cancer risk in a southern Chinese population, and validated the promising association in an eastern Chinese population. We found the SNP rs4713354A>C that is located in the 5′-untranslated region of *MDC1* was significantly associated with lung cancer risk in both populations (*P* = 0.024), with an odds ratio as 1.23(95% confidence interval  = 1.35–1.26) for the rs4713354C (CA+CC) genotypes compared to the rs4713354AA genotype. However, no significant association was observed between other SNPs and lung cancer risk. The gene-based analysis rested with these SNPs suggested the *MDC1* as a susceptible gene for lung cancer (*P* = 0.009). Moreover, by querying the gene expression database, we further found that the rs4713354C genotypes confer a significantly lower mRNA expression of MDC1 than the rs4713354AA genotype in 260 cases of lymphoblastoid cells (*P* = 0.002). Our data suggested that the SNP rs4713354A>C of *MDC1* may be a functional genetic biomarker for susceptibility to lung cancer in Chinese.

## Introduction

DNA damage response (DDR) is a sophisticated cellular procedure involving multiple molecules to repair DNA damage and maintain the genome integrity and fidelity. Usually, DNA damage can be caused by tobacco carcinogens or ionizing radiation, or other sources, it triggers DDR including activation of cell cycle checkpoint, commencement of transcriptional programs, and execution of DNA repair, or initiation of apoptosis when the damage is severe [1]–[3]. Failure to repair DNA lesions would result in genomic instability and a variety of genetically inherited disorders, such as cancer. DDR can protect the cellular DNA from damage by recruiting a series of DDR proteins that act as sensors, transducers, mediators and effectors in DDR. The DDR cascade starts with the sensors that detect the damage and transport the initial signal to the transducers. The transducers, aided by the mediators, amplify the signal and transmit it to the effectors, which carry out diverse roles such as repair, checkpoint activation and if necessary-apoptosis [4].

Mediator of DNA damage checkpoint protein 1 (MDC1), also known as Nuclear Factor with BRCT Domains 1 (NFBD1), is an important player in the DDR that regulates the activation of the intra-S phase and G2/M phase cell cycle checkpoints in response to DNA damage [5], [6]. MDC1 majorly functions as a mediator in the DDR, which mediates the recruitment of other DDR proteins, such as ataxia telangiectasia-mutated (ATM), Breast Cancer 1, Early Onset (BRCA1), Mre11/Rad50/NBS1 (MRN) complex, to the site of damage [7]–[10]. Recent evidences also showed that MDC1 has a direct role in repairing DNA double-strand breaks (DSBs) by participating in the two major DNA repair pathways, the homologous recombination and non-homologous end-joining response [11]–[13], and in the activation of the decatenation checkpoint21 and mitosis [14], [15]. Dysfunction of MDC1 has been reported to cause multiple disorders [16], [17], such as defective spermatogenesis [18]. Nowadays, more and more evidences supported MDC1 to be a potential tumor suppressor with roles in repairing DNA damage and inhibiting tumor growth [19]–[24]. MDC1 was found to be expressed lowly in various cancers including lung cancer, breast carcinomas [25] and gastric carcinoma [26].

Human *MDC* gene is located at the Chromosome 6p21.3, a region that has been reported to be a susceptible region of lung cancer in Asians by a genome-wide association study (GWAS) [27]. Previous studies have found that genetic variants of *MDC1* were associated with Epstein-Barr virus (EBV) antibody titers in Chinese and radiosensitivity in American [28], [29]. EBV and radiosensitivity are two high risk factors of human cancer, therefore, these genetic variants of *MDC1* may also affect the susceptibility of cancer. However, study on this aspect is still lacking. Single nucleotide polymorphisms (SNPs) that are located in the promoter or exons of genes have potencies on affecting gene expression or function, and thus influence the susceptibility of human diseases [30]–[32]. In the current study, we tested the hypothesis that these putatively functional SNPs of *MDC1* were associated with risk of lung cancer based on a two-stage case-control study, and assessed the function of promising SNPs by bioinformatics analysis.

## Materials and Methods

### Ethics Statement

This study was approved by the institutional review boards of Guangzhou Medical University (Ethics Committee of Guangzhou Medical University: GZMC2007–07–0676) and Soochow University (Ethics Committee of Soochow University: SZUM2008031233). All participants were scheduled for a face to face interview after written informed consents were obtained.

### Study subject

After got the approbation of the institutional review boards of Guangzhou Medical University and Soochow University, we conducted two independent case-control studies in southern Chinese and eastern Chinese, respectively. As described in previously published studies [33]–[35], 1056 histopathologically confirmed lung cancer cases and 1056 healthy controls that were frequency-matched with cases on age (±5) and sex, were collected in Guangzhou city and surrounding area; and 503 lung cancer cases and 623 frequency-matched controls were recruited in Suzhou city. The southern Chinese population was used as a discovery set, while the eastern Chinese was used as a validation set. All participants were scheduled for a face to face interview after written informed consents were obtained. They were asked to provide data on age, sex, smoking status, pack-years smoked, drinking status and family history of cancer with a structured questionnaire, as well as a 5-ml peripheral blood sample. The definitions of smoking status, pack-years smoked, drinking status and family history of cancer have been described in previously published studies [33]–[35].

### SNP selection and genotyping

We used the FuncPred block of the SNPinfo Web Server (http://snpinfo.niehs.nih.gov/) to select putatively functional SNPs of *MDC1* with a common frequency (i.e., minor allele frequency, MAF >5%) in Chinese. We found and chose five SNPs meeting the aforementioned criterion. They were rs4713354A>C (+39A>C: locating in the 39 position of the cDNA sequences), rs9262152G>A (Arg268Lys: causing an amino acid change from Arginine to Lysine at codon 268), rs2075015G>A (Glu371Lys: causing an amino acid change from Glutamic acid to Lysine at codon 371), rs28986465C>T (Pro386Leu: causing an amino acid change from Proline to Leucine at codon 386), rs9461623T>C (Ser1180Pro: causing an amino acid change from Serine to Proline at codon 1180). We genotyped above five SNPs using the Taqman allelic discrimination Assay on the ABI7900HT system (Applied Biosystems by Life Technologies, Foster City, CA) with primers and probes designed by the Primer Express 3.0 software (Applied Biosystems by Life Technologies). The primers and probes for each SNP were presented in **Table S1** in File S1.

### Statistical analysis

The frequency distribution of each SNP genotypes and Hardy-Weinberg equilibrium (HWE) of SNPs in controls, were tested by the chi-square test. The odds ratio (OR) and 95% confidence interval (95%CI) implicating association between each SNP of *MDC1* and risk of lung cancer were calculated using the unconditional logistic regression model with or without adjustment for age, sex, smoking status, drinking status and family history of cancer. The gene-based association was tested using the VEGAS software [36]. Interaction between promising SNPs and selected factors was assessed by the multiplicative interaction analysis [37]. The homogeneity of the results in two sets and in sub-groups was tested by the Breslow-Day test. Furthermore, the statistical power was calculated by using the PS Software [38]. All tests were two-sided by using the SAS software (version 9.2; SAS Institute, Cary, NC). *P*<0.05 was considered to be statistically significant.

## Results

### Association between *MDC1* SNPs and lung cancer risk

Table 1 shows the frequency distribution of the five SNPs in cases and controls. The genotype distributions of all SNPs in the controls of southern Chinese were all in agreement with the Hardy-Weinberg equilibrium (*P*>0.05 for all). Of the five SNPs, only the genotypes of rs4713354A>C exerted a significant difference in frequency distribution between the cases and controls in the discovery set (*P* = 0.006). As shown, compared with individuals carrying the common rs4713354AA genotype, those carrying the rs4713354CA genotype and rs4713354CC genotype existed 1.32-folds (odds ratio [OR] = 1.32, 95% confidence interval [95%CI] = 1.08–1.61) and 1.96-folds (OR = 1.96, 95%CI = 1.03–3.61) in risk of lung cancer, respectively. After combined the two risk genotypes, the rs4713354C variant genotypes (i.e., CA+CC) conferred a significant increase in risk of lung cancer (OR = 1.36, 95%CI = 1.12–1.65). The above associations were further verified in the eastern Chinese and the results were consistent (Berslow-Day test: *P* = 0.768) as shown in Table 1. The frequency distribution of rs4713354C genotypes was higher in cases than controls in the validation set (33.2% vs. 27.3%). The genotype frequency difference was approaching significant (*P* = 0.098). Meanwhile, the rs4713354C variant genotypes contributed to a significant increase for lung cancer risk (OR = 1.32, 95%CI = 1.02–1.71) in comparison to the rs4713354AA genotype. We then merged the two populations to increase the study power. We found that individuals carrying the rs4713354C variant genotypes had 1.33-folds increased risk of lung cancer compared with those carrying the rs4713354AA genotype (OR = 1.33, 95%CI = 1.14–1.55). The gene-based association analysis further revealed that the *MDC1* gene to be associated with lung cancer risk with an approaching statistical significance (*P* = 0.057) based on the results from above five SNPs, and the most significant associated-SNP was rs4713354A>C (*P* = 0.003). In addition, the frequency distributions of demographic characteristics of the discovery set and validation set are shown in **Table S2** in File S1.

**Table 1:** Distribution of genotypes of *MDC1* and associations with the risk of lung cancer.

Genotypes/Alleles	Case n (%)	Controls^a^ n (%)	*P* value^b^	Crude OR (95%CI)	Adjusted OR (95%CI)^c^
Discovery set					
Total no. of subjects	1056	1056			
rs4713354A>C					
AA	750(71.0)	811(76.8)	**0.006**	1.00 (ref.)	1.00 (ref.)
CA	278(26.3)	229(21.7)		**1.31(1.07–1.61)**	**1.32(1.08–1.61)**
CC	28(2.7)	16(1.5)		**1.89(1.02–3.53)**	**1.93(1.03–3.61)**
CA+CC	306(29.0)	345(23.2)		**1.35(1.11–1.64)**	**1.36(1.12–1.65)**
rs9262152G>A					
GG	915(86.6)	932(88.2)	0.357	1.00 (ref.)	1.00 (ref.)
AG	138(13.1)	119(11.3)		1.18(0.91–1.53)	1.17(0.90–1.52)
AA	3(0.3)	5(0.5)		0.61(0.15–2.27)	0.62(0.15–2.62)
rs2075015G>A					
GG	922(87.3)	911(86.3)	0.226	1.00 (ref.)	1.00 (ref.)
AG	132(12.5)	138(13.1)		0.95(0.73–1.22)	0.97(0.75–1.25)
AA	2(0.2)	7(0.6)		0.28(0.06–1.36)	0.29(0.06–1.39)
rs28986465C>T					
CC	899(85.1)	919(87.0)	0.449	1.00 (ref.)	1.00 (ref.)
TC	146(13.8)	128(12.1)		1.17(0.90–1.50)	1.16(0.90–1.49)
TT	11(1.1)	9(0.9)		1.25(0.52–3.03)	1.25(0.51–3.03)
rs9461623T>C					
TT	901(85.3)	908(86.0)	0.339	1.00 (ref.)	1.00 (ref.)
CT	154(14.6)	144(13.6)		1.08(0.84–1.38)	1.08(0.85–1.38)
CC	1(0.1)	4(0.4)		0.25(0.03–2.26)	0.26(0.03–2.37)
Validation set					
Total no. of subjects	503	623			
rs4713354A>C					
AA	336(66.8)	453(72.7)	0.098	1.00 (ref.)	1.00 (ref.)
CA	150(29.8)	153(24.6)		**1.32(1.01–1.72)**	1.31(1.00–1.72)
CC	17(3.4)	17(2.7)		1.35(0.68–2.68)	1.36(0.68–2.72)
CA+CC	167(33.2)	170(27.3)		**1.32(1.03–1.71)**	**1.32(1.02–1.71)**
Merge set					
Total no. of subjects	1559	1679			
rs4713354A>C					
AA	1086(69.7)	1264(75.3)	**0.001**	1.00 (ref.)	1.00 (ref.)
CA	428(27.4)	382(22.7)		**1.30(1.11–1.53)**	**1.30(1.11–1.53)**
CC	45(2.9)	33(2.0)		**1.58(1.01–2.51)**	**1.63(1.03–2.57)**
CA+CC	473(30.3)	415(24.7)		**1.33(1.14–1.55)**	**1.33(1.14–1.55)**

a The genotype distributions of above SNPs in controls were all in Hardy-Weinberg equilibrium (*P*>0.05).

b The frequency distribution of genotypes of SNPs between cases and controls by the chi-square test.

c Adjusted in a logistic regression model that included age, sex, smoking status, drinking status, and family history of cancer.

### Stratification analysis of the association between rs4713354A>C and lung cancer risk

**Table 2** shows the the frequency distributions of rs4713354A>C genotypes in cases and controls and associations between the SNP and lung cancer risk in each sub-group stratified by the confounding factors. No significant association between the SNP rs4713354A>C and lung cancer risk was observed in individuals with pack-years smoked <20 or ≥20 and in individuals with a history of cancer. However, this may be due to the limited sample size because the homogeneity test indicated that there was no significant difference between these stratum-ORs in each sub-group (*P*>0.05 for all). Moreover, no significant interaction was observed for the selected factors and the SNP on increasing lung cancer risk (*P*>0.05 for all), which might be due to a lack of study power for interaction analysis. In addition, results from the multivariable logistic regression analysis showed that smoking and the risk genotype of SNP rs4713354A>C were still associated with increased risks of lung cancer as shown in **Table 3** (*P*<0.001 for both).

**Table 2:** Stratification analysis of the *MDC1* rs4713354A>C genotypes by selected variables in cases and controls.

	Patients (*n* = 1559)		Controls (*n* = 1679)		Adjusted OR (95% CI)^a^	*P* b	*P* c
	CA+CC *n* (%)	AA *n* (%)	CA+CC *n* (%)	AA *n* (%)	CA+CC vs AA		
Age (years)							
≤60	245(30.3)	564(69.7)	227(25.9)	650(74.1)	1.24(1.00–1.54)	0.387	0.338
>60	228(30.4)	522(69.6)	188(23.4)	614(76.6)	1.46(1.16–1.83)		
Sex							
Male	331(30.3)	760(69.7)	299(25.2)	886(74.8)	1.29(1.07–1.55)	0.584	0.559
Female	142(30.3)	326(69.7)	116(23.5)	378(76.5)	1.44(1.08–1.92)		
Smoking status							
Ever	244(29.6)	580(70.4)	194(25.4)	571(74.6)	1.24(1.00–1.55)	0.366	0.089
Never	229(31.2)	506(68.8)	221(24.2)	693(75.8)	1.43(1.14–1.77)		
Pack-years smoked							
≥20	175(28.0)	449(72.0)	118(24.6)	361(75.4)	1.20(0.91–1.57)	0.564	0.339
<20	69(34.5)	131(65.5)	76(26.6)	210(73.4)	1.43(0.97–2.12)		
0	229(31.2)	506(68.8)	221(24.2)	693(75.8)	1.43(1.14–1.77)		
Drinking status							
Ever	93(31.7)	200(68.3)	80(23.4)	262(76.6)	1.47(1.02–2.17)	0.390	0.463
Never	380(30.0)	886(70.0)	335(25.1)	1002(74.9)	1.29(1.08–1.53)		
Family history of cancer							
Yes	31(24.0)	98(76.0)	37(26.1)	105(73.9)	0.90(0.51–1.29)	0.147	0.131
No	442(30.9)	988(69.1)	378(24.6)	1159(75.4)	1.38(1.17–1.62)		
Histological types			415(24.7)	1264(75.3)			
Adenocarcinoma	194(31.5)	421(68.5)			1.40(1.14–1.72)	0.831	
Squamous cell carcinoma	159(30.2)	368(69.8)			1.31(1.06–1.64)		
Large cell carcinoma	21(31.8)	45(68.2)			1.41(0.83–2.40)		
Small cell lung cancer	56(29.0)	137(71.0)			1.25(0.90–1.74)		
Other carcinomas	38(26.4)	106(73.6)			1.08(0.73–1.59)		
Stages							
I	63(31.5)	137(68.5)	415(24.7)	1264(75.3)	1.40(1.02–1.93)	0.683	
II	39(26.5)	108(73.5)			1.09(0.74–1.59)		
III	156(31.8)	334(68.2)			1.42(1.14–1.77)		
IV	215(29.8)	507(70.2)			1.29(1.06–1.57)		

a ORs were adjusted for age, sex, smoking status, drinking status, and family history of cancer.

b *P* value of Breslow-Day test.

c *P* value of test for the multiplicative interaction.

**Table 3:** A multivariable logistic regression analysis for lung cancer risk.

Variable	ß	SE	OR	95%CI	*P*
age	−0.004	0.004	1.00	0.99–1.00	0.208
sex	0.198	0.125	1.22	0.95–1.56	0.113
Smoking status	0.475	0.086	**1.61**	**1.36–1.90**	**<0.001**
Drinking status	−0.012	0.131	0.99	0.77–1.28	**0.927**
Family history of cancer	−0.054	0.128	0.95	0.74–1.22	0.674
rs4713354A>C	0.285	0.079	**1.33**	**1.14–1.55**	**<0.001**

### Genotype-phenotype correlation by bioinformatics analysis

The SNP rs4713354A>C is located at the 5′-untranslated region (5′-UTR) of *MDC1* gene, it may affect the transcript activity of *MDC1* promoter. We therefore performed bioinformatics analyzes to explore the possible function of this SNP on MDC1 expression. By querying the Snpexp database (http://app3.titan.uio.no/biotools/tool.php?app=snpexp), we found a significant correlation between the rs4713354A>C genotypes and mRNA expression levels of MDC1 in 260 cases of lymphoblastoid cells in all population under the dominant genetic model (*P* = 0.002). Cells carrying the rs4713354C variant genotypes expressed significantly lower mRNA levels of MDC1 (CA: 9.066±0.184; CC: 9.030±0.185) than cells carrying the rs4713354AA genotype (9.138±0.237). We further used the SNPinfo Web server (http://snpinfo.niehs.nih.gov/) to predict the possible molecular mechanism of this SNP on affecting gene expression and found that the A to C transvertion of rs4713354A>C would result in a loss of binding sites of three transcription factors (TFs) that are CEBPA, CEBP and NR2F2.

## Discussion

Multiple evidences supported that the *MDC1* gene to be a potential tumor suppressor resting with its essential roles in repairing DNA damage and its interactions with several important tumor-related genes, such as P53, NBS1 and 53BP1 [19]–[24], [39], [40]. Here, we found that the SNP rs4713354A>C of *MDC1* was associated with risk of lung cancer in Chinese. The rs4713354C variant genotypes could cause a low expression of MDC1 *in vivo* and thus contributed to an increased lung cancer risk. However, we did not find any significant associations between other four putatively functional SNPs of *MDC1* and lung cancer risk. Further analysis supported the *MDC1* gene to be a susceptible gene and rs4713354A>C to be a susceptible loci of lung cancer. To the best of our knowledge, this is the first report on genetic variants of *MDC1* and susceptibility of cancer.

Aberrant reduction or lack of MDC1 was observed in lung cancer tissues [25], and down-regulation of MDC1 expression in lung cancer cells would result in defective radiation-induced apoptosis [41]. Moreover, the toxin cantharidin can cause DNA damage by inhibiting MDC1 expression in lung cancer cells [42]. Thus, loss expression of MDC1 is an important condition during lung carcinogenesis. The SNP rs4713354A>C is located at the 5′-UTR of *MDC1*, a region generally recognized as promoter or exonic splicing element of genes. Bioinformatics analyses showed that the A to C transvertion of rs4713354A>C causes a loss of binding sites of three TFs that are CEBPA, CEBP and NR2F2, and the rs4713354C variant genotypes exert a decreased MDC1 expression *in vivo*. This is consistent in biological plausibility with our observation of rs4713354C variant genotypes conferred an increased risk of lung cancer. Interestingly, not only the three TFs involves lung cancer development [43]–[45], but also CEBPA plays an important role in cell cycle [46]. It is possible that the two molecules, CEBPA and MDC1 might have a cross-talk on regulating cell cycle, which needs to further study.

A few studies have investigated the association between *MDC1* SNPs and risk of human diseases. However, the results were controversial. One synonymous variant of *MDC1* was reported to be associated with increased radiosensitivity but not prostate cancer risk [29]. An Chinese study reported a variant allele of *MDC1* exhibited a significant association with EBV seropositivity [28]. A Caucasian study reported no variant of *MDC1* was associated with breast cancer risk as well as DNA-damaging effects of radiation therapy [47]. However, the above studies were all lack of study power because of their limited sample sizes. In the current study, based on a two-stage case-control study with a relatively large sample size, we showed that a promoter SNP of *MDC1* contributed to a significant increased risk of lung cancer in Chinese. The study power was strong in the current study, as we achieved a 94.47% study power (two-sided test, α = 0.05) to detect an OR of 1.33 for the rs4713354C variant genotypes, which occurred at a frequency of 24.7% in the controls. Further analysis based on the results from the five putatively functional SNPs of *MDC1* suggested *MDC1* to be a susceptible gene for lung cancer.

Since our study was a hospital-based case-control study, it had some limitations such as bias, including selection bias and information bias. These may cause spurious associations between the studied SNPs and cancer risk. However, four points supported our results were not achieved by chance and the significant association was credible. The first was that we have achieved two consistent results in two independent populations. The second was that we have achieved a strong study power. The third was that the bioinformatics analyses demonstrated a consistence in biological plausibility with our observation. In addition, results from the Chinese GWAS also showed that the frequency of rs4713354A>C genotypes was different between cases and controls with approaching statistical significance (*P* = 0.078) [48].

In conclusion, our data showed that the promoter SNP rs4713354A>C of *MDC1* and the *MDC1* gene were associated with lung cancer risk in Chinese by influencing MDC1 expression. Both the SNP rs4713354A>C and *MDC1* might be a genetic biomarker for susceptibility of lung cancer in Chinese. Validations with larger population based studies in different ethnic groups are warranted.

## Supporting Information

File S1 (DOC)

Table S1 **The primers and probes for the five putatively functional SNPs of MDC1.**
(DOC)

Table S2 **Frequency distributions of selected variables in cases and controls.**
(DOC)
