# Correction: A Newfound Association between *MDC1* Functional Polymorphism and Lung Cancer Risk in Chinese

**DOI:** 10.1371/journal.pone.0117882

**Published:** 2015-01-30

**Authors:** The PLOS ONE Staff

There are errors in the values of the fourth and sixth sentences in the Abstract. The correct fourth sentence is: We found the SNP rs4713354A>C that is located in the 5′-untranslated region of *MDC1* was significantly associated with lung cancer risk in both populations (P = 0.001), with an odds ratio as 1.33(95% confidence interval = 1.14–1.55) for the rs4713354C (CA+CC) genotypes compared to the rs4713354AA genotype. The correct sixth sentence is: The gene-based analysis rested with these SNPs suggested the *MDC1* as a susceptible gene for lung cancer (P = 0.057).
