# Supplementary material for: A Newfound Association between MDC1 Functional Polymorphism and Lung Cancer Risk in Chinese
Source: PLoS One. 2014 Sep 8;9(9):e106794. doi: 10.1371/journal.pone.0106794 (PMC4157800; doi:10.1371/journal.pone.0106794)
Supplement: File S1 — (DOC) [file pone.0106794.s001.doc]

***Table S1.*** *The primers and probes for the five putatively functional SNPs of MDC1*

| SNP | Primers | Probes *^a^* | Color (genotype) |
| --- | --- | --- | --- |
| rs4713354A>C | 5’-TGCCGGCAACGGATAGAG-3’ (forward) | FAM-TGTCTGGTA***C***GGC-MGB (alleleC) | Blue (AA) |
|  | 5’-TCTAGGAGGACCGAGGAAAGG-3’(reverse) | HEX-TGTCTGGTA***A***GGC-MGB (allele A) | Red (AC) |
|  |  |  | Green(CC) |
| rs9262152C>T | 5’-TCCCTGCACCCCTCTTGAC-3’(forward) | FAM-TTTGTATCATTGTCC***T***TC-MGB(allele G) | Blue (TT) |
|  | 5’-TAACTGAAATCCAGCTTGAAAAGGA-3’(reverse) | HEX-TTTGTATCATTGTCC***C***TC-MGB (allele A) | Red (CT) |
|  |  |  | Green(CC) |
| rs2075015G>A | 5’-CCTGGAGCACCAGGCCT-3’(forward) | FAM-CATCTGCAG***A***AGAGCCA-MGB (alleleA) | Blue (AA) |
|  | 5’-AGCACCAGGCCTGGCC-3’ (reverse) | HEX-CATCTGCAG***G***AGAGCCA-MGB (allele G) | Red (AG) |
|  |  |  | Green(GG) |
| rs9461623A>G | 5’-TCTGTGGAGGTGGAAGGCTG-3’ (forward) | FAM-AGATGTGGGCTCAG***A***GGT -MGB (allele G) | Blue (GG) |
|  | 5’-AGGGGTCTTGACAGAGGACCTAT-3’(reverse) | HEX-AGATGTGGGCTCAG***G***GGT -MGB (allele A) | Red (AG) |
|  |  |  | Green(AA) |

*^a^* Bold italic nucleotides indicate the polymorphic sites in probes.

**Table S2.** Frequency distributions of selected variables in cases and controls

| Variables | Discovery set (Southern Chinese) | | |  | Validation set (Eastern Chinese) | | |
| --- | --- | --- | --- | --- | --- | --- | --- |
|  | Case  (n=1056)  n(%) | Control  (n=1056)  n(%) | *P* ***^a^*** |  | Case  (n=503)  n(%) | Control  (n=623)  n(%) | *P* ***^a^*** |
| Age (years) |  |  |  |  |  |  |  |
| ≤ 60 | 536(50.8)  520(49.2) | 534(50.6) | 0.931 |  | 273(54.3) | 343(55.1) | 0.793 |
| > 60 |  | 522(49.4) |  |  | 230(45.7) | 280(44.9) |  |
| Sex |  |  |  |  |  |  |  |
| Male | 746(70.6) | 746(70.6) | 1.000 |  | 345(66.6) | 433(70.4) | 0.496 |
| Female | 310(29.4) | 310(29.4) |  |  | 158(31.4) | 184(29.6) |  |
| Family history of cancer |  |  |  |  |  |  |  |
| Yes | 104(9.9) | 103(9.8) | 0.942 |  | 25(5.0) | 44(7.1) | 0.046 |
| No | 952(90.1) | 953(90.2) |  |  | 478(95.0) | 579(92.9) |  |
| Family history of lung cancer |  |  |  |  |  |  |  |
| Yes | 42(4.0) | 30(2.8) | 0.150 |  | 10(2.0) | 13(2.1) | 0.907 |
| No | 1014(96.0) | 1026(97.2) |  |  | 493(98.0) | 610(97.9) |  |
| Smoking status |  |  |  |  |  |  |  |
| Current | 394(37.3) | 366(34.6) | 0.028 |  | 118(23.5) | 168(26.6) | 1.79×10^-7^ |
| Former | 207(19.3) | 176(16.8) |  |  | 105(20.9) | 57(9.2) |  |
| Never | 455(43.1) | 514(48.7) |  |  | 280(55.6) | 400(64.2) |  |
| Pack-years smoked |  |  |  |  |  |  |  |
| ≥20 | 459(43.5) | 314(29.7) | 9.40×10^-12^ |  | 165(32.8) | 165(26.5) | 0.011 |
| <20 | 142(13.4) | 228(21.6) |  |  | 58(11.5) | 58(9.3) |  |
| 0 | 455(43.1) | 514(48.7) |  |  | 280(55.7) | 400(64.2) |  |
| Drinking status |  |  |  |  |  |  |  |
| Current | 165(15.6) | 186(17.6) | 0.042 |  | 36(7.2) | 75(12.0) | 0.017 |
| Former | 64(6.1) | 41(3.9) |  |  | 28(5.6) | 40(6.4) |  |
| Never | 827(78.3) | 829(78.5) |  |  | 439(87.2) | 508(81.6) |  |
| Histological types |  |  |  |  |  |  |  |
| Adenocarcinoma | 384(36.4) |  |  |  | 231(45.9) |  |  |
| Squamous cell carcinoma | 369(34.9) |  |  |  | 158(31.4) |  |  |
| Large cell carcinoma | 43(4.1) |  |  |  | 23(4.6) |  |  |
| Small cell lung cancer | 128(12.1) |  |  |  | 65(12.9) |  |  |
| Other carcinomas *^b^* | 132(12.5) |  |  |  | 26(5.2) |  |  |
| Stages |  |  |  |  |  |  |  |
| I | 154(14.6) |  |  |  | 46(9.2) |  |  |
| II | 94(8.9) |  |  |  | 53(10.5) |  |  |
| III | 333(31.5) |  |  |  | 157(31.2) |  |  |
| IV | 475(45.0) |  |  |  | 247(49.1) |  |  |

***^a^*** *P* values for a χ^2^ test.

***^b^*** Mixed-cell or undifferentiated carcinoma.
